# ORGANIZATIONAL READINESS FOR A VIRTUAL REALITY TRAINING PROGRAM CALLED BE EPIC-VR

**DOI:** 10.1093/geroni/igad104.1051

**Published:** 2023-12-21

**Authors:** Marie Savundranayagam, Annette Schumann, Adriana Sagrak, Grace Norris, Allison Chen, Jennifer Campos, Joseph Orange

**Affiliations:** Western University, London, Ontario, Canada; Western University - Sam Katz Community Health & Aging Research Unit, London, Ontario, Canada; Sam Katz Community Health & Aging Research Unit, London, Ontario, Canada; Sam Katz Community Health & Aging Research Unit, London, Ontario, Canada; Western University - Sam Katz Community Health & Aging Research Unit, London, Ontario, Canada; University of Toronto, Toronto, Ontario, Canada; Western University, London, Ontario, Canada

## Abstract

An essential first step to implementing virtual reality programming in home care and long-term care settings is to assess organizational readiness by determining factors that enhance the likelihood of its successful implementation. Be EPIC-VR is one such virtual reality program that supports dementia-specific, person-centered communication training for frontline healthcare workers. Guided by the Consolidated Framework for Implementation Research (CFIR), the current study aimed to identify factors influencing Be EPIC-VR’s implementation in home care and long-term care settings. Semi-structured interviews were conducted with nine managers from home care and long-term care settings in Canada. Transcripts from these interviews were analyzed using the Framework Analysis’ five-step ongoing, iterative process: familiarization, identifying a thematic framework, indexing, charting, and mapping/interpretation. Textual data were open-coded and organized deductively (using CFIR’s pre-set codes) and inductively (for emergent codes) into themes and subthemes. Four themes emerged as factors contributing to organizational readiness including 1) openness to virtual reality as a training tool, 2) staffing and training logistics, 3) organizational culture supporting staff development, and 4) external pressures for organizational sustainability. These findings will guide how the Be EPIC-VR implementation team collaborates with organizational decision makers to ensure that Be EPIC-VR is a good fit for those organizations, to prepare for its implementation, and to optimize the likelihood of success. The study findings offer valuable insights for researchers and practitioners working to implement new virtual reality interventions in home care and long-term care settings.

